# ﻿Sympatry of genetic lineages of *Parisotomanotabilis* s. l. (Collembola, Isotomidae) in the East European Plain

**DOI:** 10.3897/zookeys.1137.95769

**Published:** 2022-12-21

**Authors:** Anastasia Striuchkova, Irina Malykh, Mikhail Potapov, Nataliya Kuznetsova

**Affiliations:** 1 Department of Zoology and Ecology, Institute of Biology and Chemistry, Moscow State Pedagogical University, Moscow, Russia Moscow State Pedagogical University Moscow Russia

**Keywords:** 28S rDNA, cryptic diversity, genetic lineages, microarthropods, soil fauna, springtails

## Abstract

*Parisotomanotabilis* (Schaeffer, 1896) is one of the most abundant eurytopic species of springtails in temperate regions of the northern hemisphere, and is often used as a model species for studies on the genetics of soil microarthropod populations. Six genetic lineages (L0, L1, L2, L3, L4-Saltzwedel, L4-Hebert) are known which are distributed mainly parapatrically in Western and Central Europe. Individuals of *P.notabilis* from 21 locations on the East European Plain were analyzed. Three genetic lineages were found: L1, L2, L4-Hebert. In contrast to Western and Central Europe, the coexistence of two or three lineages was revealed in about half of the locations on the East European Plain. The most diverse genetic composition of *P.notabilis* populations was noted in natural forests and slightly disturbed habitats, while the least diverse was in places with a high anthropogenic influence.

## ﻿Introduction

*Parisotomanotabilis* Schaeffer, 1896 (Collembola: Isotomidae) is a cosmopolitan species which occurs in almost every biotope in temperate regions of the Western Palearctic, and predominates in most communities of Collembola (Potapov 2001). This species is eurytopic and is moderately tolerant to disturbed habitats (Kuznetsova 2002), showing resistance to pesticides (Petersen and Krogh 1987), heavy metals (Eitminaviciute 2006; Winkler et al. 2018), application of various fertilizers (Buchholz et al. 2017) and moderate trampling (Nadezhdina and Kuznetsova 2010). One of the reasons for its success is parthenogenesis, which allows *P.notabilis* to be one of the first colonizers of disturbed habitats (Alvarez et al. 1997). Although males sometimes occur, the parthenogenetic form is found across the entire distribution range of this species (Chernova et al. 2009).

*Parisotomanotabilis* is morphologically uniform in spite of its occurrence in wide range of habitats (Potapov 1991, 2001). Genetic studies reveal heterogeneity of the species in the COI gene (Chahartaghi 2007). As such, four lineages of *P.notabilis* (L0–L3) have previously been proposed based on genetic analysis of both the COI and the D2 region of the 28S genes (Porco et al. 2012a). These results were confirmed (Saltzwedel et al. 2017) also by D3–D5 region of 28S and histone H3, and supplemented by two new genetic lineages of L4 (Hebert et al. unpub. data; Anslan and Tedersoo 2015; Saltzwedel et al. 2017). Unfortunately, the two different new lineages (see Results) were named the same “L4”. To distinguish them, we refer to the lineages as “L4-Hebert” and “L4-Saltzwedel” in the paper. The average genetic *p*-distances between lineages were very high: from 15% to 18% for the COI gene, from 5% to 11% for histone H3, and from 0.5% to 1.9% for D3–D5 region 28S (Saltzwedel et al. 2017). For comparison, the interspecies COI divergence for closely related species of Collembola ranges from 16.35% to 24.55% (Sun et al. 2018). This suggests that *P.notabilis* may have as yet undetected morphological differences that would warrant subspecies and possibly species status, if detected.

Genetic variation of *P.notabilis* suggests lineages may be parapatric as distributional data show some geographical specificity of lineages. Specifically, L1 and L2 are the most widely distributed lineages in Europe; the lineage L1 is widespread in the south and east of Europe, while L2 is found in western and northern Europe and in the Pyrenees. The L0 shows a fairly continuous range from the English Channel and along the coasts of the North and Baltic Seas. The lineage L3 has been found only in Paris and Greece (Porco et al. 2012a; Saltzwedel et al. 2017), the L4-Hebert lineage in Canada and Estonia (Hebert et al. unpub. data; Anslan and Tedersoo 2015), and the L4-Saltzwedel in Croatia (Saltzwedel et al. 2017). Coexistence of the lineages was recorded once in eastern Canada, where three lineages were mixed together (Porco et al. 2012a; Saltzwedel et al. 2017). Eastern Europe was poorly studied: only two localities have previously been examined in the territory of the European part of Russia: Karelia (L2 – 5 ind.) and the Moscow region (L1 – 5 ind.) (Saltzwedel et al. 2017). Also, the coexistence and ecological segregation of the lineages of *P.notabilis* has not been investigated. The reaction to habitat disturbance is also unknown. Cases of such segregation of genetic lineages are known, for example, in another widespread springtail species - *Lepidocyrtuslanuginosus* (L.), which differs in distribution along a disturbance gradient (Zhang et al. 2018), as well as in the *Isotomuruspalustris* group (Carapelli et al. 1995). However, it remains unclear how common the phenomenon of genetic segregation of genetic lineages is in Collembola.

In this paper we provide new data on the three lineages of *P.notabilis* in the eastern regions of Europe and we test two hypotheses: 1) whether the genetic lineages of *P.notabilis* in the Eastern Europe are distributed parapatrically, as in Western Europe; and 2) whether different genetic lineages of *P.notabilis* react differently to habitat disturbance.

## ﻿Material and methods

### ﻿Sample collection

The study was conducted in the central region of the East European Plain, mainly around Moscow, which includes a wide range of habitats from natural forests to urban lawns. The territory is located in a belt of mixed and broad-leaved forests. The climate is temperate continental, the average annual amplitude of temperature variation is 28 °C, and 600–800 mm of precipitation falls per year (Kulbachevskii 2021). Three types of habitats representing different disturbance were selected: natural forests (10 locations), forest parks (six locations), urban lawns (five locations). One 2-litre sample of the litter and / or topsoil was taken from 1 × 1 m area in each location. Samples were taken from 21 locations from October 2020 to February 2021 (Appendix 1). Springtails were extracted into 96% alcohol using Tullgren funnels, without heating, until the samples were completely dry (7-10 days).

The extracted material was sorted under a stereomicroscope. The possible mixing with coexisting congeners of *P.notabilis* was considered. Apart from *P.notabilis*, four species of the genus *Parisotoma* have been recorded in the East European Plain: *P.agrelli* Delamare Deboutteville, 1950, *P.ekmani* Fjellberg, 1977, *P.reducta* Rusek, 1984, and *P.trichaetosa* Martynova, 1977; all four species are rare and only the latter species was recorded in the Moscow region previously. In appearance all four species are easy to separate from *P.notabilis* by having an almost white corpus and smaller eye spots. The first three congeners occur only in northern areas and are rarely recorded in the central region of the East European Plain. The littoral *P.agrelli* lives only on the Arctic Ocean shore, while the Asiatic *P.reducta* is distributed in the very north-east corner the East European Plain. The boreal *P.ekmani* can probably occur towards the central part of the East European Plain via peat-bogs although it was never recorded there in spite of intensive study of the region (its distribution is given in more detail in Potapov 1991, 2001). *Parisotomatrichaetosa* was formally recorded in the Moscow region (Potapov et al. 2021). It is an invasive Asiatic species (Potapov and Janion-Scheepers 2019) with two known single records in the area under study. *Parisotomatrichaetosa* is distinct by its overall appearance, quadridentate mucro, and many other characters. The differentiating characters of *P.notabilis* and its congeners mentioned above are given in Table 1. Apart from *P.notabilis*, other species of *Parisotoma* were not recorded in our samples. Our preliminary sorting of specimens of *P.notabilis* was confirmed by the identification by the ribosomal 28S gene region. Besides, the specimens from several samples of each lineage were mounted on slides and identified using existing identification keys (Potapov 2001; Fjellberg 2007).

**Table 1.** Key differentiating morphological characters of species of *Parisotoma* recorded in the East European Plain. Abbreviations: Omma: number of ommatidia, Postlab: number of postlabial setae, VT: number of laterodistal setae on ventral tube, Subcx: presence of outer seta on 2-nd subcoxa of first pair of legs, Mucro: number of teeth on mucro, s: number of s-setae on tergites.

Species	Omma	Postlab	VT	Subcx	Mucro	s
*P.notabilis*	3–4	4+4	3+3	-	3	complete
*P.agrelli*	1	3+3	3+3	+	3	complete
*P.ekmani*	1	4+4	4+4	-	3	reduced
*P.reducta*	1	3+3	3+3	+	3	complete
*P.trichaetosa*	1	4+4	3+3	-	4	reduced

### ﻿Molecular analyses

The lineages of this species can be identified by both the mitochondrial COI gene and the ribosomal 28S gene (D2 region, Porco et al. 2012a; D3–D5 region, Saltzwedel et al. 2017). We used only the D3–D5 region of 28S which showed higher positive PCR products. Ninety-seven individuals from 21 locations were selected for analysis. DNA extraction was performed using the Thermo Scientific Phire Tissue Direct PCR Master Mix. DNA was extracted from single individuals in 20 μl DNA Dilution Buffer and 0.5 μl DNA Release Additive and incubated at 98 °C for 2 min. This technique allows DNA extraction with relatively little damage to the original material, enabling vouchers to be deposited. A 573 bp D3–D5 fragment of the nuclear 28S rDNA was amplified using the primers 28Sa 5’-GAC CCG TCT TGA AGC ACG-3’ and 28Sbout 5’-CCC ACA GCG CCA GTT CTG CTT ACC-3’ (Whiting 2002; Prendini et al. 2005). COI was amplified using the primers ColFol-for 5’-TTT CAA CAA ATC ATA ARG AYA TYG G-3’ and ColFol-rev 5’-TAA ACT TCN GGR TGN CCA AAA AAT CA-3’ (Ramirez-Gonzalez et al. 2013). This data was used to correlate the results with L4-Hebert, for which there are no data on D3–D5 fragment of 28S. To a master mix consisting of 7 µl of nuclease-free Water, 10 µl of Phire Tissue Direct PCR Master Mix, 1 µl of forward and reverse primers were added 1 µl of individual DNA. PCR conditions included one initial activation step at 98 °C for 5 min, followed by 30 amplification cycles of denaturation at 98 °C for 5 s, annealing at 57 °C (28S) or 55 °C (COI) for 5 s, elongation at 72 °C for 20 s and a final elongation step at 72 °C for 1 min. The result of the PCR was evaluated by electrophoresis in agarose gel with ethidium bromide. PCR products were purified using an enzyme mix of 0.5 µl of exonuclease I (Exo I) and 1 µl of recombinant alkaline phosphatase (rSAP) per 5 µl of PCR product, according to the protocol: 37 °C for 15 min and 80 °C for 15 min. The purified product was dried with the addition of the forward primer. Sequencing was performed in the Synthol laboratory. The sequences obtained were edited in Chromas Lite (v. 2.6.6) (http://technelysium.com.au/wp/chromas/). Sequences were then aligned using BioEdit (v. 7.2) (Hall et al. 2011). Tree construction and calculation of genetic distances between lineages (K2P-pairwise distances) were performed using the MEGA-X program (v.11) (Tamura et al. 2021). Tree calculation was performed with the Maximum Likelihood method with the Jukes-Cantor + Gamma Distributed parametric model proposed by MEGA-X (lowest BIC = 4531.333, AICc = 2599.369) for the 28S gene, and the Neighbor-Joining method with Tamura 3-parameter + Gamma Distributed parametric model for the COI gene. Sequences were obtained for 87 individuals for a D3–D5 fragment of the 28S gene 573 bp and 12 individuals for a COI gene 619–657 bp, which are additional confirmation of the results. The tree for the 28S gene is based on our data and those of Saltzwedel et al. (2017). GenBank (www.ncbi.nlm.nih.gov/GenBank) accession numbers are listed in the Appendix 1. Detail data on genetic lineages records were placed in the international Global Biodiversity Information Facility (GBIF) in the ‘sampling event dataset’ format (Striuchkova et al. 2022).

## ﻿Results

### ﻿Genetic lineages

**Figure 1.**
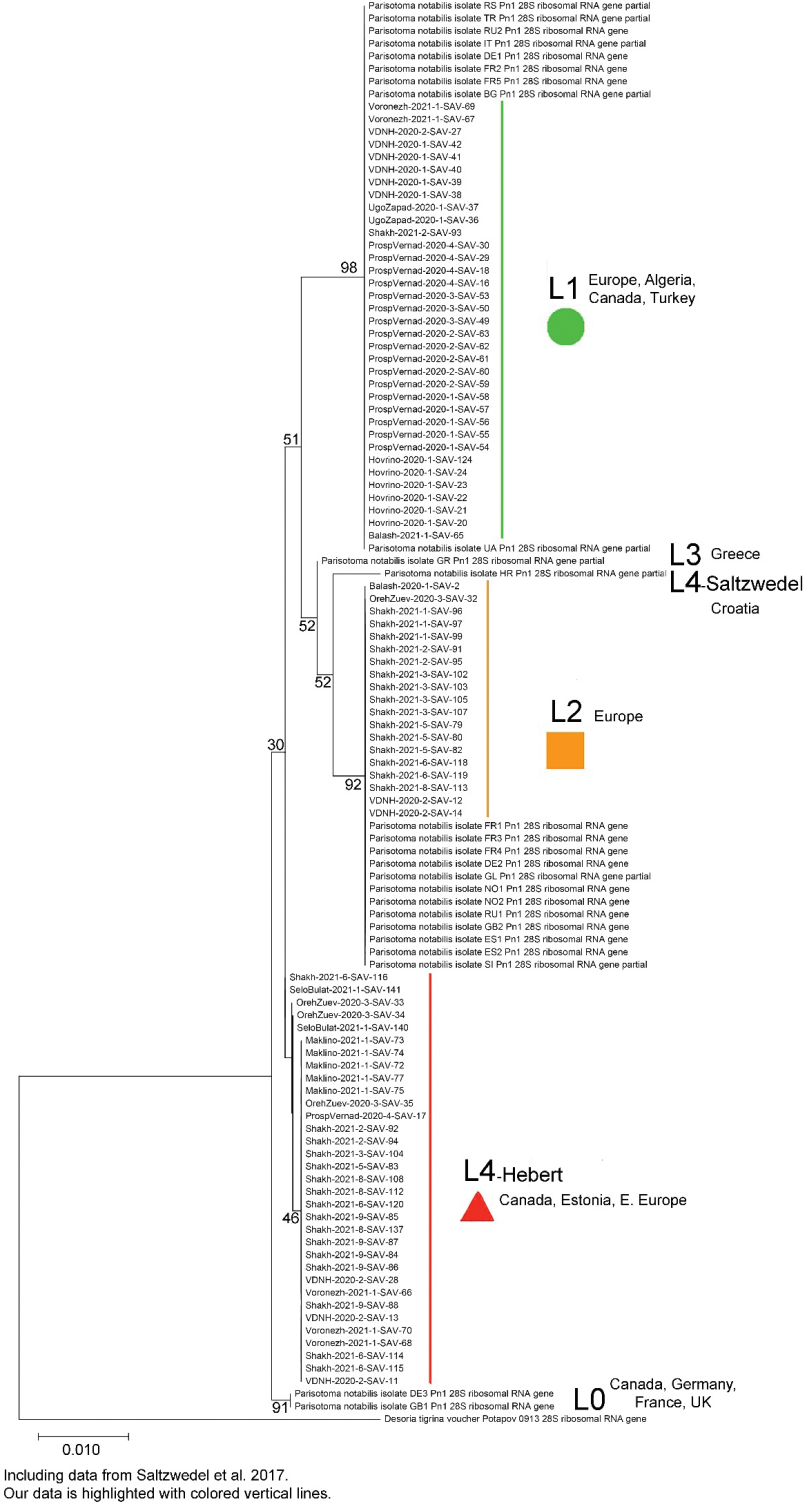
Maximum Likelihood genetic tree of six lineages of *P.notabilis* based on the D3-D5 region of 28S gene fragment (Bootstrap support values shown on the branches, scale bar shows genetic distance) including data from Saltzwedel et al. (2017) availed in GenBank. Our data is highlighted with colored vertical lines.

The constructed tree revealed three genetic lineages of *P.notabilis*: L1, L2, and L4-Hebert (Fig. 1). The last one is not represented for D3–D5 region of 28S in GenBank. It was identified by COI according to blast results in the BOLD database (Hebert et al. unpub. data, Anslan and Tedersoo 2015). Genetic distances (observed K2P-distances in %) between the lineage L4-Hebert (this study) and L4-Saltzwedel for COI gene = 17.6%. The genetic distances between the L1, L2 and L4-Hebert lineages for the D3–D5 region of 28S ranged from 0.9% to 1.4% in our material. Mean genetic distances between all known lineages and intralineage distances are given in Table 2. Neighbor-Joining genetic tree and intralineage and interlineage K2P-pairwise distances for the COI gene are presented in Appendices 2 and 3, respectively.

**Table 2.** Intralineage and interlineage K2P-pairwise distances (%) of the lineages *Parisotomanotabilis* in the East European Plain for D3–D5 region of 28S gene.

Lineage	Intralineage	Interlineage				
		L0	L1	L2	L3	L4-Saltzwedel
L1	0	1.23	0			
L2	0	1.23	1.41	0		
L3	0	0.70	0.89	0.53	0	
L4-Saltzwedel	0	1.59	1.77	1.06	0.88	0
L4-Hebert	0	0.50	0.90	1.03	0.50	1.06

### ﻿Coexistence of genetic lineages

**Figure 2.**
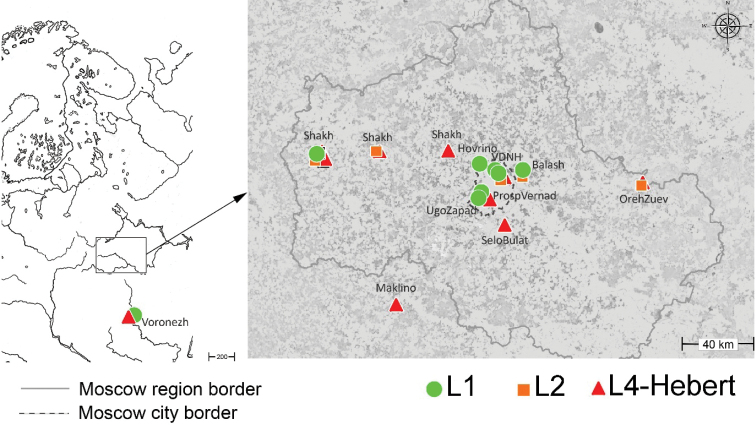
Records of the genetic lineages of *Parisotomanotabilis* (green circle - L1, orange square - L2, red triangle – L4-Hebert). See Appendix 1 for location data for each lineage.

Numerous cases of sympatry of the lineages were revealed (Fig. 2). Two lineages were found in one third of the locations (41%), with three lineages in 12% of the locations. Almost half (47%) of the locations contained only one lineage. Table 3 shows the ratios of the number of sites with lineage sympatry / number of total sites sampled based on the literature (Porco et al. 2012a; Saltzwedel et al. 2017) and our data.

**Table 3.** Local diversity of genetic lineages in different regions of Europe.

Region	Total number of individuals	Total number of locations	Ratio	Reference
Western and Central Europe	191	27	0.18*	Porco et al. 2012a**
	110	24	0.08	Saltzwedel et al. 2017***
East European Plain	87	21	0.5*	This study

* excluding locations with 1 or 2 individuals. ** excluding data from Canada and Algeria. *** excluding data from Russia

### ﻿Ecological specificity of the lineages L1, L2 and L4-Hebert

**Figure 3.**
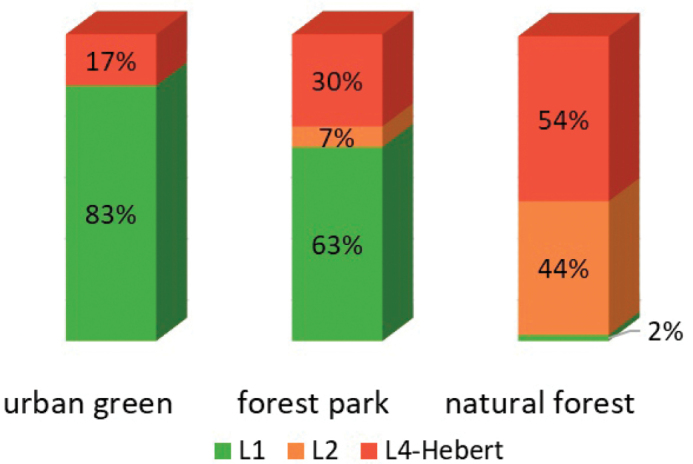
Records of genetic lineages of *P.notabilis* (L1, L2 and L4-Hebert) in different groups of habitats, % of the number of samples in the habitats of the group.

L1 and L4-Hebert lineages were recorded in the urban areas, and all three lineages - in the partly disturbed (forest parks) and undisturbed (forests) habitats. In the forests, the occurrence of L2 and L4-Hebert lineages was about the same, while L1 was sporadic. In forest parks, L1 was the most common, the L4-Hebert was less common, while L2 was rare. In the city (urban areas), L1 absolutely dominated (Fig. 3).

## ﻿Discussion

### ﻿Genetic lineages

The L1 and L2 lineages of *P.notabilis* were already cited in the East European Plain (Saltzwedel et al. 2017). The lineage L4-Hebert has been identified based on the sequences COI available in GenBank (Hebert et al. unpub. data; Anslan and Tedersoo 2015). The L4-Hebert for D4–D5 region of 28S is most similar with the L0 and L3 lineages (0.50%) which have not yet found in the East European Plain. The L4-Hebert lineage showed the greatest differences with the L1, L2, and L4-Saltzwedel lineages (0.90–1.05%). Updated data in the present study agree with the previously estimated average genetic distance between the most common lineages of *P.notabilis* L1 and L2 in Europe and Canada (1.4% vs. 1.39% according to Saltzwedel et al. 2017).The L4-Hebert lineage is probably widespread in the East European Plain, because we recorded it also in Voronezh, which is about 500 km south of Moscow.

### ﻿Coexistence of genetic lineages

Previously, studies on the genetic structure of *P.notabilis* populations showed mainly parapatric distribution of lineages. The simultaneous presence of two or three lineages was noted only in 13% of the total number of locations studied (Porco et al. 2012a; Saltzwedel et al. 2017). Consequently, the sympatry of the lineages in Canada was thought to be possible independent introductions of the species, that is, they consider the case likely to be accidental (Porco et al. 2012a). In our data, the sympatry of the lineages showed markedly more frequency than in the west - almost in half of the locations studied (Table 3). The ratio number of sites with lineages sympatry / number of total sites sampled is greater in our findings. In Western and Central Europe this value is 0.8–0.18, while in the East European Plain it is 0.5. The joint occurrence of lineages was noted not only in different areas of the Moscow region, but also in Voronezh. In Eastern Europe, sympatry of the lineages L2 and L4-Hebert was also found in Estonia (Anslan and Tedersoo 2015).

**Figure 4.**
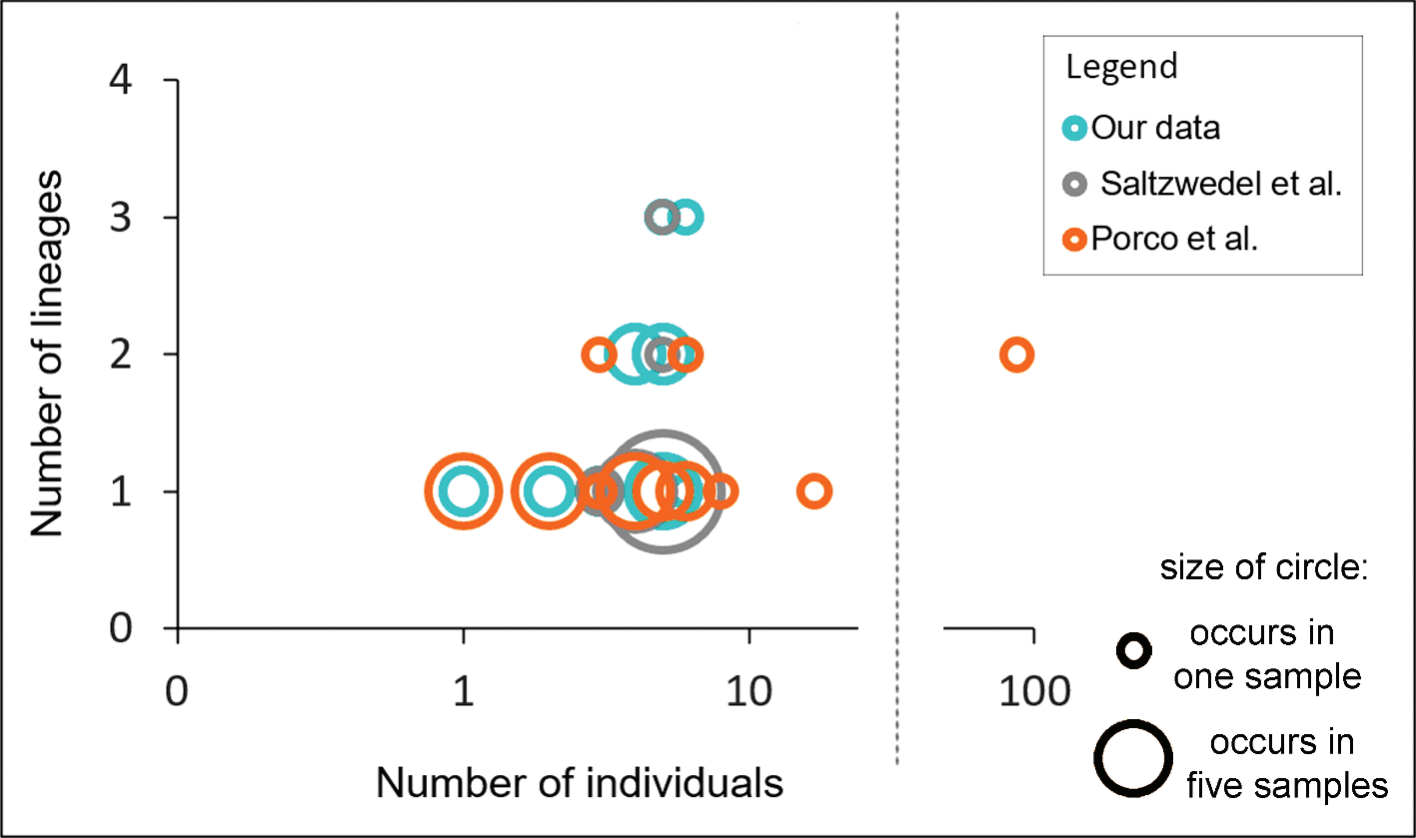
Dependence of the number of detected *P.notabilis* lineages on the number of individuals analyzed in a sample in Europe. The larger the circle, the more samples contain a certain combination of the number of lineages and the number of individuals.

Presumably, the number of detected lineages in one location may depend on the number of individuals analyzed from a core. Our data shows, however, that even small samples of 4 or 5 individuals sometimes revealed up to three genetic lineages (Fig. 4), while a sample of 17 individuals in Hamburg (Germany) was represented by only one lineage (Porco et al. 2012a). Our material is collected over a relatively small area compared to extensive data from Western Europe. It is possible that the urban condition contributes to the conservation of invasive species (Rebele 1994) and also should concern genetic lineages. Nevertheless, it is hard to ignore that several lineages are found in true natural forests (Shakh-2021-2) located more than 100 km away Moscow city. It is likely that the relatively separated lineages of *P.notabilis* in Western and Central Europe do co-occur in the east. One of the reasons for this phenomenon may be the geologically young postglacial landscape of the region we studied. This flat area had no refugia and broke free from ice only a few tens of thousands of years ago (Velichko et al. 2004) and so it is still an arena of active migratory flows and evolutionarily young communities, which is especially evident in the sedentary groups of organisms (Markova et al. 2008). The species introduced into such young communities are more successful than those introduced into ancient communities. A similar explanation can supplement the discussion of the reasons for the sympatry of *P.notabilis* lineages in Canada as a result of their introduction to North America (Porco et al. 2012a; Saltzwedel et al. 2017). Our hypothesis on parapatry of the lineages in the East European Plain has not been confirmed.

At present, it is difficult to conclude how widespread sympatry of genetic lineages is. For example, sympatry of genetic lineages was found in *Deutonuramonticola* (Cassagnau) and *Heteromurusmajor* (Moniez) among 16 genetically studied Collembola species (Porco et al. 2012b).

### ﻿Ecological specialization of lineages

Genetic studies of springtails often do not consider the habitats where the material was collected from, or, at least, do not specify them. Saltzwedel et al. (2017) preliminarily supposed that environmental conditions may select for different lineages of *P.notabilis*. We investigated the genetic composition of *P.notabilis* populations not only in natural but also in disturbed habitats. The results showed an association of the L1 lineage with disturbed habitats but L2 and L4-Hebert with natural habitats. This indicates possible differences in tolerance to disturbance among genetic lineages.

The different habitat preferences of genetic lineages reflect the process of ecological diversification within one species, and can lead to the emergence of new species resulting from ecological speciation. For many taxa, such cases were noted based on the action of different selection vectors according to the gradient speciation model (Doebeli and Dieckmann 2003; Endler 2020). This phenomenon was found, for example, in the “*palustris*” group of the genus *Isotomurus*, the genetic analysis of which made it possible to distinguish six species within *I.palustris* (Mueller) *sensu lato* distributed in different sections of the river floodplain profile (Carapelli et al. 1995). Subsequently, broad genetic variability was discovered in many Collembola species, but the cryptic diversity of this taxon was mostly described in terms of genetic lineages rather than independent species (Porco et al. 2012a). Obviously, more genetic, morphological, and ecological information must be accumulated to standardize the criteria for distinguishing Collembola species.

Information on the ecological preferences of genetic lineages is sporadic. Thus, in the widespread springtail species *Lepidocyrtuslanuginosus* the genetic lineage L1 was abundant and occurred in each of the three habitats studied (forests, grassland, arable fields), L2 only in forests, and L3 only in pastures and arable fields (Zhang et al. 2018). A similar diversification is found in *P.notabilis*: one lineage is unspecialized to habitats (L4-Hebert), while two are more specialized, one to natural forests (L2), the other to disturbed habitats (L1). In both species the environmental factors leading to lineage specialization remain unclear. The adaptations of the lineage may be of ecophysiological, nutritional, demographic or migrational nature.

Our results are preliminary and call for more data. However, the assumption that different genetic lineages of *P.notabilis* prefer different degrees of habitat disturbance seems convincing at this point. Samples along the disturbance gradient can reveal the diversity of lineages for one location. The cosmopolitan species *P.notabilis*, abundant in natural and disturbed habitats, is a promising model object for studying the phylogeography of Collembola populations.

## ﻿Conclusions

Sequencing of the 28S gene is practical and convenient to identify already known genetic lineages while data on the COI gene are needed to describe new lineages. Three genetic lineages of *Parisotomanotabilis* have been detected in the East European Plain: L1, L2, and L4-Hebert. Mean genetic distances between the lineages in the studied D3–D5 region of ribosomal 28S gene region ranged from 0.9% to 1.4%. About half of the samples in central part of East European Plain included more than one lineage of *P.notabilis*. The samples from different habitats may include different genetic lineages of the species, which is important to take into account in phylogeographic reconstructions. The most diverse genetic composition of *P.notabilis* populations was observed in natural forests and forest parks; only two lineages were found in urban environments. Genetic lineages of *P.notabilis* show ecological specialization: L1 likely prefers disturbed habitats, although L2 and L4-Hebert predominate in natural forests, which requires further research. It is also necessary to focus on the search for characters that could allow the morphological differentiation of the lineages.

## Figures and Tables

**Figure A1.**
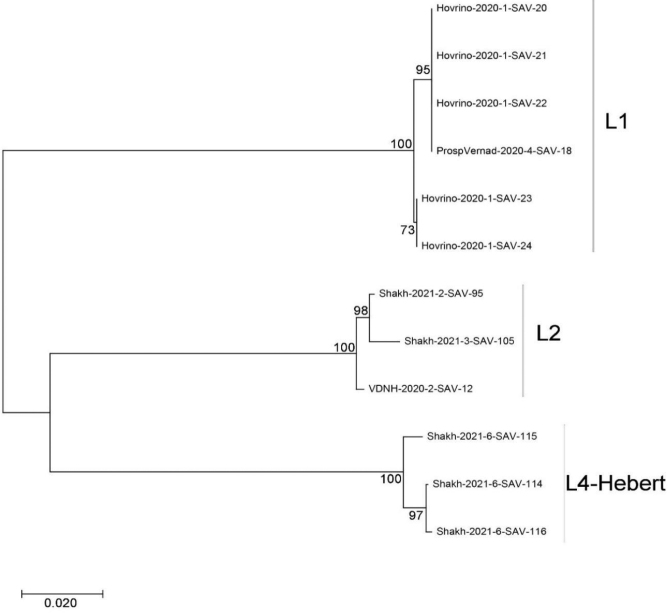
Neighbor-Joining genetic tree of three lineages of *P.notabilis* based of COI gene fragment (Bootstrap support values showed on the branches, bar is genetic distance).

## ﻿Appendix 1

**Table A1.** Sample locations and GenBank Accession numbers of genetic lineages of *Parisotomanotabilis*.

Habitat	Label	GPS Coordinates	N	Lineage	GenBank Accession number	
					28S	COI
Urban green	Balash-2021-1*	55°49'54.1069'N, 37°58'2.7689'E	1	L1	OM714597	
	ProspVernad-2020-1	55°40'54.2149'N, 37°30'22.6072'E	5	L1	OM746101–OM746105	
	UgoZapad-2020-1*	55°39'34.8097'N, 37°28'46.7176'E	2	L1	OM778178, OM778179	
	VDNH-2020-1	55°48'58.5949'N, 37°39'0.3845'E	5	L1	OM778173–OM778177	
	Voronezh-2021-1	51°39'33.9962'N, 39°12'7.1375'E	2	L1	OM778153, OM778154, OM778158–OM778160	
			3	L4-Hebert		
Forest Park	Hovrino-2020-1	55°52'24.7849'N, 37°28'42.3077'E	6	L1	OM728286–OM728291	OP861639–OP861643 (L1)
	ProspVernad-2020-2	55°41'8.5393'N, 37°30'4.5928'E	5	L1	OM746085–OM746089	
	ProspVernad-2020-3	55°41'9.5113'N, 37°29'46.7008'E	3	L1	OM746096–OM746098	
	ProspVernad-2020-4	55°40'53.4301'N, 37°29'59.8372'E	4	L1	OM746081–OM746084, OM746095	OP866972 (L1)
			1	L4-Hebert		
	VDNH-2020-2	55°48'48.6229'N, 37°39'55.4105'E	1	L1	OM778143, OM778144, OM778150–OM778152, OM778181	OP861659 (L2)
			2	L2		
			3	L4-Hebert		
	Maklino-2021-1	54°59'43.8770'N, 36°27'25.7931'E	5	L4-Hebert	OM746090–OM746094	
Nature forest	Balash-2020-1*	55°49'52.4545'N, 37°54'22.9853'E	1	L2	OM714532	
	OrehZuev-2020-3	55°46'43.2060'N, 39°16'13.9757'E	1	L2	OM745895, OM746106–OM746108	
			3	L4-Hebert		
	SeloBulat-2021-1*	55°31'1.9789'N, 37°40'38.7112'E	2	L4-Hebert	OM746099, OM746100	
	Shakh-2021-1	55°56'33.8150'N, 35°31'45.7780'E	3	L2	OM778155–OM778157	
	Shakh-2021-2	55°55'55.7450'N, 35°37'9.4468'E	1	L1	OM778148, OM778149, OM778169–OM778171	OP861657 (L2)
			2	L2		
			2	L4-Hebert		
	Shakh-2021-3	55°56'0.2990'N, 35°37'20.9560'E	4	L2	OM778164–OM778168	OP861658 (L2)
			1	L4-Hebert		
	Shakh-2021-5	55°59'1.6022'N, 35°35'41.5816'E	3	L2	OM778145–OM778147, OM778180	
			1	L4-Hebert		
	Shakh-2021-6	55°59'2.4266'N, 35°35'50.4664'E	2	L2	OM757828–OM757831, OM778140, OM778141	OP861662–OP861664
			4	L4-Hebert		(L4-Hebert)
	Shakh-2021-8	55°59'30.6829'N, 36°14'57.8500'E	1	L2	OM778142, OM778161–OM778163	
			3	L4-Hebert		
	Shakh-2021-9	55°59'14.9005'N, 37°2'57.3605'E	5	L4-Hebert	OM778135–OM778139	

*Not used in calculating the number of lineages per location. *N*: number of specimens **GenBank Accession number of Saltzwedel et al. (2017) used in building the tree (Fig. 1.): KJ792225, KJ792230, KJ792235, KJ792240, KJ792244, KJ792249, KJ792254, KJ792263, KJ792268, KJ792272, KJ792277, KJ792282, KJ792287, KJ792291, KJ792295, KJ792299, KJ792304, KJ792309, KJ792314, KJ792319, KJ792324, KJ792327, KJ792332, KJ792335, KJ792340.

## ﻿Appendix 3

**Table A2.** Intralineage and interlineage K2P-pairwise distances (%) of tree lineages *Parisotomanotabilis* in the East European Plain for COI gene.

Lineage	Intralineage	Interlineage	
		L1	L2
L1	0.20	0	
L2	0.76	19.84	0
L4-Hebert	0.59	20.74	17.33

## References

[B1] Alvarez T Frampton GK Goulson D (1997) Population dynamics of epigeic Collembola in arable fields: the importance of hedgerow proximity and crop type. Pedobiologia 41: 110–114. http://sro.sussex.ac.uk/id/eprint/51314/ [November 10, 2022]

[B2] Anslan S Tedersoo L (2015) Performance of cytochrome c oxidase subunit I (COI), ribosomal DNA Large Subunit (LSU) and Internal Transcribed Spacer 2 (ITS2) in DNA barcoding of Collembola. European Journal of Soil Biology 69: 1–7. 10.1016/j.ejsobi.2015.04.001

[B3] Buchholz J Querner P Paredes D Bauer T Strauss P Guernion M Scimia J Cluzeau D Burel F Kratschmer S Winter S Potthoff M Zaller JG (2017) Soil biota in vineyards are more influenced by plants and soil quality than by tillage intensity or the surrounding landscape. Scientific Reports 7(1): e17445. 10.1038/s41598-017-17601-w PMC5727173 29234045

[B4] Carapelli A Frati F Fanciulli PP Dallai R (1995) Genetic differentiation of six sympatric species of *Isotomurus* (Collembola, Isotomidae); is there any difference in their microhabitat preference? European Journal of Soil Biology (France). https://agris.fao.org/agris-search/search.do?recordID=FR9606618 [September 26, 2021]

[B5] Chahartaghi M (2007) Trophic niche differentiation, sex ratio and phylogeography of European Collembola. phd. Technische Universität. http://elib.tu-darmstadt.de/diss/000850 [June 10, 2021]

[B6] Chernova NN Potapov MB Savenkova Y Bokova A (2009) Ekologicheskaya Rol’ Partenogeneza u Kollembol. Zoologicheskii Jurnal 88: 1455–1470. https://naukarus.com/ekologicheskaya-rol-partenogeneza-u-kollembol [November 10, 2022]

[B7] Doebeli M Dieckmann U (2003) Speciation along environmental gradients. Nature 421(6920): 259–264. 10.1038/nature01274 12529641

[B8] Eitminaviciute I (2006) Microarthropod communities in anthropogenic urban soils. 1. Structure of microarthropod complexes in soils of roadside lawns. Entomological Review 86(S2): S128–S135. 10.1134/S0013873806110029

[B9] Endler JA (2020) Geographic variation, speciation and clines. (MPB-10), Volume 10. Princeton University Press. 10.12987/9780691209456 409931

[B10] Fjellberg A (2007) 42 The Collembola of Fennoscandia and Denmark, Part II: Entomobryomorpha and Symphypleona. Brill, Leiden. 10.1163/ej.9789004157705.i-265 [November 10, 2022]

[B11] Hall T Biosciences I Carlsbad C (2011) BioEdit: An important software for molecular biology. GERF Bulletin of Biosciences 2: 60–61.

[B12] Kulbachevskii AO (2021) Doklad o sostoyanii okrujayuschei sredi v gorode Moskve v 2020 godu. Moscow, 330 pp. https://www.mos.ru/eco/documents/doklady/view/259642220/ [February 28, 2022]

[B13] Kuznetsova N (2002) Biotopic groups of Collembolans in the mixed forest subzone of Eastern Europe. Entomological Review 82: 1047–1057. https://istina.msu.ru/publications/article/128149331/ [November 10, 2022]

[B14] Markova A Kolfschoten T Bohnke S Kosinsev PA Mol J Puzachenko A Simakova AN Smirnov N Verpoorte A Golovachev IV (2008) Evolution of European Ecosystems during Pleistocene-Holocene Transition (24–8 Kyr BP). KMK Scientific Press, Moscow. [November 10, 2022]

[B15] Nadezhdina TS Kuznetsova NA (2010) The influence of recreational load on soil-dwelling collembolans in different forest associations. Entomological Review 90(4): 415–422. 10.1134/S0013873810040020

[B16] Petersen H Krogh PH (1987) Effects of perturbing microarthropod communities of a permanent pasture and a ryefield by an insecticide and a fungicide. In: Soil fauna and soil fertility. Moscow, 217–229.

[B17] Porco D Potapov M Bedos A Busmachiu G Weiner WM Hamra-Kroua S Deharveng L (2012a) Cryptic Diversity in the Ubiquist Species *Parisotomanotabilis* (Collembola, Isotomidae): A Long-Used Chimeric Species? PLoS ONE 7(9): e46056. 10.1371/journal.pone.0046056 PMC3458803 23049931

[B18] Porco D Bedos A Greenslade P Janion C Skarżyński D Stevens MI van Vuuren BJ Deharveng L Porco D Bedos A Greenslade P Janion C Skarżyński D Stevens MI van Vuuren BJ Deharveng L (2012b) Challenging species delimitation in Collembola: Cryptic diversity among common springtails unveiled by DNA barcoding. Invertebrate Systematics 26(6): 470–477. 10.1071/IS12026

[B19] Potapov M (1991) Species of the genus IsotomasubgenusParisotoma Bagnall, 1940 and Sericeotoma subgen. nov. (Collembola, Isotomidae) of USSR fauna. Acta Zoologica Cracoviensia 34: 267–301. http://www.isez.pan.krakow.pl/journals/azc/pdf/azc_i/34(1)/34(1)_06.pdf [November 10, 2022]

[B20] Potapov M (2001) Synopses on Palaearctic Collembola: Isotomidae. Abhandlungen und Berichte des Naturkundemuseums Gorlitz 73: 1–603. https://www.nhbs.com/synopses-on-palaearctic-collembola-volume-3-isotomidae-book [November 10, 2022]

[B21] Potapov M Janion-Scheepers Ch (2019) Longitudinal invasions of Collembola within the Palearctic: new data on non-indigenous species. In: Abstracts of 10^th^ International Seminar on Apterygota. Paris, France, 46–46. https://isa10.sciencesconf.org/data/pages/ISA10_2019_Programme_Final.pdf [November 10, 2022]

[B22] Potapov M Kuznetsova NA Janion-Scheepers C Bokova AI Panina KS (2021) Alien species of Collembola in agroecosystems in the european part of Russia. In: Invasion of alien species in Holarctic. Borok-VI., 184.

[B23] Prendini L Weygoldt P Wheeler WC (2005) Systematics of the *Damonvariegatus* group of African whip spiders (Chelicerata: Amblypygi): Evidence from behaviour, morphology and DNA. Organisms, Diversity & Evolution 5(3): 203–236. 10.1016/j.ode.2004.12.004

[B24] Ramirez-Gonzalez R Yu DW Bruce C Heavens D Caccamo M Emerson BC (2013) PyroClean: Denoising pyrosequences from protein-coding amplicons for the recovery of interspecific and intraspecific genetic variation. PLoS ONE 8(3): e57615. 10.1371/journal.pone.0057615 PMC3585932 23469211

[B25] Rebele F (1994) Urban ecology and special features of urban ecosystems. Global Ecology and Biogeography Letters 4(6): 173–187. 10.2307/2997649

[B26] Saltzwedel H Scheu S Schaefer I (2017) Genetic structure and distribution of *Parisotomanotabilis* (Collembola) in Europe: Cryptic diversity, split of lineages and colonization patterns. PLoS ONE 12(2): e0170909. 10.1371/journal.pone.0170909 PMC5295681 28170395

[B27] Striuchkova A Potapov M Kuznetsova N Malykh I (2022) Genetic lineages of *Parisotomanotabilis* s. l. (Collembola, Isotomidae) in the East European Plain. Version 1.3. Moscow Pedagogical State University (MPSU). Sampling event dataset. 10.15468/5rm9kz [accessed via GBIF.org on 2022-12-07] PMC9836473 36760483

[B28] Sun X Bedos A Deharveng L (2018) Unusually low genetic divergence at COI barcode locus between two species of intertidal *Thalassaphorura* (Collembola: Onychiuridae). PeerJ 6: e5021. 10.7717/peerj.5021 PMC6011825 29938135

[B29] Tamura K Stecher G Kumar S (2021) MEGA11: Molecular Evolutionary Genetics Analysis Version 11. Molecular Biology and Evolution 38(7): 3022–3027. 10.1093/molbev/msab120 33892491 PMC8233496

[B30] Velichko AA Faustova MA Gribchenko YN Pisareva VV Sudakova NG (2004) Glaciations of the East European Plain-distribution and chronology. Developments in Quaternary Science 2: 337–354. 10.1016/S1571-0866(04)80083-6

[B31] Whiting MF (2002) Mecoptera is paraphyletic: Multiple genes and phylogeny of Mecoptera and Siphonaptera. Zoologica Scripta 31(1): 93–104. 10.1046/j.0300-3256.2001.00095.x

[B32] Winkler D Bidló A Bolodár-Varga B Erdő Á Horváth A (2018) Long-term ecological effects of the red mud disaster in Hungary: Regeneration of red mud flooded areas in a contaminated industrial region. The Science of the Total Environment 644: 1292–1303. 10.1016/j.scitotenv.2018.07.059 30743842

[B33] Zhang B Chen T-W Mateos E Scheu S Schaefer I (2018) Cryptic species in *Lepidocyrtuslanuginosus* (Collembola: Entomobryidae) are sorted by habitat type. Pedobiologia 68: 12–19. 10.1016/j.pedobi.2018.03.001

